# MicroRNA expression profiling of endocrine sensitive and resistant breast cancer cell lines

**DOI:** 10.1016/j.bbrep.2022.101316

**Published:** 2022-07-20

**Authors:** Maitham A. Khajah, Alyaa Al-Ateyah, Yunus A. Luqmani

**Affiliations:** Faculty of Pharmacy, Kuwait University, Safat, 13110, Kuwait

**Keywords:** MicroRNAs, Breast cancer, Endocrine resistance, EMT, miR-449a, miR-29a-3p, miR-200c-3p

## Abstract

**Background:**

MicroRNAs (miRs) regulate gene expression through translation inhibition of target mRNAs. One of the most promising approaches for cancer therapy is through mimicking or antagonizing the action of miRs. In this report, we analyzed the miRnome profile of several human breast cancer cell lines to determine the influence of estrogen receptor (ER) silencing previously shown to result in epithelial to mesenchymal transition (EMT) and enhanced tumor invasion.

**Methods:**

MicroRNA extracted from MDA-MB-231 (*de novo* ER-) and ER-silenced (*acquired* ER-) pII and IM-26 or ER-expressing (YS1.2) siRNA transfected derivatives of MCF7 cells was deep sequenced on Illumina NextSeq500. Respective miRnomes were compared with edgeR package in R and Venny2.1 and target prediction performed with miRTarBase. Mimics and inhibitors of selected differentially expressed miRs associated with EMT mediators (miR-200c-3p targeting ZEB1, miR-449a targeting δ-catenin and miR-29a-3p) were transfected into pII cells and mRNA targets, as well as E-cadherin and keratin 19 (epithelial and mesenchymal markers respectively) were measured using taqman PCR.

**Results:**

Each cell line expressed about 20% of the total known human miRnome; There was a high degree of similarity between the 3 tested ER-lines. Out of these expressed miRs, 50–60% were significantly differentially expressed between ER- and ER + lines. Transfection of miR-200c-3p mimic into pII cells down regulated ZEB1 and vimentin, and increased E-cadherin and keratin 19 with accompanying morphological changes, and reduced cell motility, reflecting a reversal back into an epithelial phenotype. On the other hand, transfecting pII with miR-449a inhibitor reduced cell invasion but did not induce EMT. Transfecting pII cell line with the mimic or inhibitor of miR-29a-3p showed no change in EMT markers or cell invasion suggesting that the EMT induced by loss of ER function can be reversed by blocking some but not just any random EMT-associated genes.

**Conclusions:**

These data suggest that differences in miR expression can be exploited not only as mediators (using mimics) and targets (using miR antagonists) for general cancer therapies aimed at regulating either individual or multiple mRNAs, but also to re-sensitize endocrine resistant breast cancers by turning them back into a type that will be susceptible to endocrine agents.

## Highlights

• Around 50–60% of microRNAs were significantly differentially expressed between ER- and ER + breast cancer cell lines.
• Transfection of miR-200c-3p mimic into ER -ve cells induced MET and reduced cell motility.
• Transfecting of miR-449a inhibitor into ER -ve cells reduced cell invasion but did not induce EMT.

## 1 Introduction

MicroRNAs (miRNAs) are a class of single-stranded, non-protein coding RNAs with an approximate length of 19–25 nucleotides. They act through binding to the 3′ un-translated regions (UTR) of target messenger RNAs (mRNAs) through complementarity with the first 2–8 nucleotides at the 5′ end of the miRNA, affecting over 30% of the human genome [1,2]. Numerous reports have documented the importance of miRNAs in various physiological and pathophysiological conditions [[3], [4], [5], [6], [7], [8], [9]]. In relation to cancer, miRNAs are involved in the pathogenesis of various forms of cancers through regulating the activity of intracellular signaling molecules such as MAPK, PI3K/PTEN, NFƙB, TGFβ, Notch, and Hedgehog, which are involved in controlling multiple processes including proliferation, apoptosis and angiogenesis [[10], [11], [12], [13]]. Single nucleotide polymorphisms (SNP) in various miRNAs have been linked to predisposition of various cancers [[14], [15], [16]] including of the breast [17]. There are many reports of altered expression of miRNAs. For example, miRNA-21 demonstrated enhanced expression profile, while the expression of miRNAs-126, -143, and −145 was reduced in most (∼80%) types of tumors [18]. Metastatic breast tumors show elevated miRNA-10b and reduced miRNA-126, -206, and −335 levels [19,20]; this was shown to be associated with longer relapse-free survival [21]. High expression of *let-7*, miRNAs-21, -23, and −27a has been linked with drug resistance in ovarian cancer [22], whereas miRNA-452 was shown to be significantly down-regulated in adriamycin-resistant breast cancer cells; targeting insulin-like growth factor-1 receptor (IGF-1R) [23].

Accumulating *in vitro* data suggests that epithelial to mesenchymal transition (EMT) [24,25] plays a significant role in breast cancer pathogenesis and is regarded as a key hallmark feature of cancer; a recent review by Mittal et al. [26] presents the current evidence from *in vivo* studies. A number of miRNAs that either induce or inhibit the EMT process in breast cancer have been identified (*Luqmani Y and Khajah M 2015, MicroRNA in Breast Cancer - Gene Regulators and Targets for Novel Therapies, A Concise Review of Molecular Pathology of Breast Cancer, Mehmet Gunduz, IntechOpen, DOI: 10.5772/59428*). For example, miRNA-9 is up-regulated in breast cancers relative to normal tissues [27], and in primary breast tumors from patients with diagnosed metastases, in comparison with those from metastasis-free patients [28]. Ectopic expression of miRNA-9 induced EMT-like conversion in human mammary epithelial cells *in vitro* with a significant decrease in the epithelial marker E-cadherin and increase in the mesenchymal marker vimentin expression [28]. Expression of miRNA-24 was significantly increased in breast cancer cell lines which had undergone TGF-β-induced EMT as well as in metastatic tumors compared with primary breast tumor samples with mesenchymal phenotype. The induction of EMT through miRNA-24 was in part through targeting the guanine nucleotide exchange factor Net1A; an activator of Rho kinase [29,30]. The expression of miRNA-29 [31], miRNA-29a [32], miRNA-103/107 [33,34], miRNA-106b-25 cluster [35], miRNA-155 [36], and miRNA-221/222 [37] was also increased in invasive mesenchymal-like breast cancer cell lines and their over-expression in non-invasive breast cancer cells induced EMT and enhanced cell invasion. Other types of miRNAs have been shown to inhibit/reverse the EMT process. The expression of miRNA-7 [38], miRNA-124 [39], miRNA-145 [40], miRNA-200 family [41], miRNA-205, miRNA-375 [42], and miRNA-448 [43] was decreased in invasive breast cancer cells and their inhibition in non-invasive cells induced the EMT process through targeting of various molecules including STAT3, SLUG, Oct-4, ZEB1/2, SNAIL and PI3K/Akt.

In our laboratory, we have established a cellular model of EMT in breast cancer cells which developed in parallel with endocrine resistance induced by blockade of estrogen receptor (ER)-α mRNA translation in the parental MCF-7 cells using ER-directed shRNA transfection. Several cell lines established from such transfections are all characterized by enhanced expression of mesenchymal markers (e.g. vimentin and *N*-cadherin), reduced expression of epithelial markers (such as E-cadherin), morphological change to spindle-like shape, and enhanced cellular proliferative, motile and invasive capacity [[44], [45], [46], [47]]. We have previously shown that epidermal growth factor was the most potent activator of endocrine resistant cell invasion through enhanced Akt and ERK1/2 phosphorylation, and matrix metalloproteinase activity [46]. It is thought that cancer cells which have undergone this transformation into a mesenchymal-like cell actually revert back to their original epithelial character during the process of establishing as a metastatic niche [48,49]. We see this reversion as a therapeutic opportunity to reduce or prevent primary metastasis. It was therefore of interest to investigate the expression profile of miRNA in our two cell types to identify potential miRNAs that could be manipulated for this purpose. We performed miRnome sequencing analysis for various ER-positive and -negative (both *acquired* and *de novo* resistant) breast cancer cells aiming to obtain an overall expression profile of miRNAs in relation to EMT/endocrine resistance. As a test case, we specifically targeted miR-449a and miR-200c in the endocrine resistant pII cell line to determine whether it was possible to reverse any aspect of the EMT process which had already occurred in these cells. As indicators we examined epithelial/mesenchymal markers, cell morphology and invasive capacity.

## 2 Materials and methods

### 2.1 Cell culture

MCF-7 (ER +) and MDA-MB-231 (ER-) human breast carcinoma cell lines were obtained from the ATCC (American Type Culture Collection, VA, USA). pII and IM-26 cell lines were established in this laboratory by transfection of MCF-7 with ER directed shRNA silencing plasmid as described previously (Supplementary Table 1) [45,50].

YS1.2 was derived from a transfection with ER shRNA plasmid that *failed* to down-regulate ER; this is used as a transfected control ER expressing cell line. All cell lines were maintained as monolayers at 37 °C in an incubator gassed with an atmosphere of 5% CO_2_ at 95% humidity and cultivated in Advanced Dulbecco's Minimum Essential Medium (DMEM) supplemented with 5% fetal bovine serum (FBS), 600 μg/ml l-glutamine, 100 U/ml penicillin, 100 μg/ml streptomycin and 6 ml/500 ml 100 x non-essential amino acids (all from Invitrogen, CA, USA).

### 2.2 miRNA deep sequencing

Cultures of all tested cell lines were grown to approximately 80% confluency in 75 cm flasks, detached by trypsinisation, centrifuged for 5 min at 600 g, washed with 1 ml PBS and centrifuged again for 5 min at 600 g. Cell pellets (approximately 7 × 10 [6] cells) were then re-suspended in 100 μL of PBS and 600 μL of RNAlater (Sigma-Aldrich) to stabilize the RNA and stored at 4 °C prior to extraction. RNA was isolated using the miRNAeasy mini kit (Qiagen) following the manufacturer's instructions. On-column DNase digestion was performed during the RNA extraction. The RNA concentration was determined using the NanoDrop 2000 UV–Vis spectrophotometer (Thermo Scientific). RNA quality control was performed using the 2100 Bioanalyzer microfluidic gel electrophoresis system (Agilent). Three independent samples from each cell line were sent for sequencing analysis to Biogazelle NV, Zwijnaarde, Belgium. They constructed libraries for small RNA sequencing using the TruSeq small RNA library kit (Illumina) according to the manufacturer's instructions. Briefly, 100 ng of total RNA was used as input for RNA adapter ligation (using 3′ and 5′ RNA adapters) followed by reverse transcription and PCR amplification with bar-coded primers. PCR products were separated on a Pippin Prep system (Sage Science) to recover the 147 nt and 157 nt fractions containing mature miRNAs. Small RNA libraries were sequenced on a NextSeq500 instrument from Illumina. Reads were filtered based on stringent read quality control. After adapter trimming, reads were collapsed and mapped to the genome using Bowtie [51]. Mapped reads were subsequently annotated to different contaminants (tRNA, rRNA, sn(o)RNA, piRNA) and mature miRNAs using genome annotation data from Ensembl, UCSC and miRBase v20. On average, 20.1 million reads were generated per sample, with a minimal read count of 9.29 million reads. Read length distribution and annotation was evaluated per sample to ensure enrichment of miRNAs in the 20–24 nt read fraction.

Prior to normalization, data were filtered using a cutoff of 4 reads (i.e., only those miRNAs with or more reads were considered expressed). miRNA expression data were normalized based on the total read count per sample. Read count for each miRNA was divided by the total read count in that sample and multiplied by the median total read count across all samples. After normalization, data were log2-transformed. Sample-sample clustering (Euclidean distance, complete agglomeration) was performed on pairwise Pearson correlation coefficients based on the miRNA level read counts or the normalized miRNA expression data. Differential gene expression analysis was performed separately by pairwise comparisons. For each analysis, only miRNAs with a read count >4 in at least three samples were retained for further analysis. Differential gene expression analysis was performed on the raw read count data with the edgeR package in R which is a software for examining differential expression of replicated count data from Bioconductor [52]. Volcano plots were generated by plotting the log2FC versus -log10 (P-value) of each comparison. Heat-maps were generated in R based on the normalized miRNA expression levels of the top 50 differentially expressed miRNAs. For functional enrichment analysis, first the predicted targets of all miRNAs were downloaded from miRDB 5.0 [53]. Next, gene sets associated with the terms ”Estrogen receptor”, ”EMT”, ”invasion”, ”motility” and ”metastasis” were retrieved from the Molecular Signature Database [54]. Finally, for each miRNA, a Fisher Exact test was performed to assess the enrichment of the genes in a certain gene set, among its predicted targets. Subsequently, per miRNA, Benjamini-Hochberg multiple testing correction was performed [55]. Gene-sets were pre-selected from the Molecular Signature Database that were associated (Juan Carlos Oliveros. n.d. “Venny2.1).with the following biological processes:” Estrogen receptor”,” EMT”,” invasion”,” motility”,” metastasis” and” cell adhesion”. Predicted target genes for each of the candidate miRNAs were retrieved via miRDB 5.0. Finally, multiple Fisher exact test was performed to assess the over-representation of a miRNA's target genes in each of the pre-selected gene sets.

Venn diagrams were constructed using the Venny2.1 software (Juan Carlos Oliveros n.d. “Venny2.1) in order to compare between the ER-cell lines MDA-MB-231, pII and IM26, and with the ER + cell line YS1.2. The average of three triplicates was taken except for when two out of three had no expression and the third value was less than 3 then this miRNA was not considered, otherwise even if the mean was less than 4 the miRNA was considered for the Venn diagram. Members that were exclusively expressed in YS1.2, MDA-MB-231, all ER-cell lines or all ER-acquired resistant cell lines were considered only if significantly differentially expressed in all comparisons. Targets of miRNAs and members in the miRNA families that were differentially expressed between ER- and ER + cells were predicted using miRTarBase [56], and only experimentally validated targets were considered. Targets of miRNAs of *de novo* resistant cell line MDA-MB-231 and acquired resistance cell lines pII and IM26 were compared and predicted using the miRTarBase and analyzed as mentioned before.

### 2.3 RNA extraction and cDNA synthesis

For measurements of gene expression, total RNA was extracted using the RNeasy kit from Qiagen, (USA). Purification was carried out according to the manufacturers' instructions. Quantity and quality were measured using NanoDrop 1000 spectrophotometer (Thermo Scientific) and by 2100 Bioanalyzer microfluidic gel electrophoresis system (Agilent Technologies, Inc). The purified RNA samples were stored in RNAse-free distilled water at −80 °C. cDNA was synthesized from 1 μg of total RNA using High-Capacity cDNA Reverse Transcription Kit in the presence of RNase inhibitor 2000U (both from ABI, USA) following the manufacturers’ instructions. The PCR amplification was carried out on a thermal cycler (ABI, USA) with the following parameters: 25 °C for 10 min, 37 °C for 120 min, 85 °C for 5 min.

### 2.4 Quantitative real-time PCR

Levels of Dicer, CTNND2 and ZEB1 mRNAs were measured using corresponding TaqMan Gene Expression Assays (ABI, USA) according to the manufacturer's instructions. The PCR cycling was carried out in a 7600 Fast real time instrument (ABI) under the following conditions: 50 °C (2 min) hold, 95 °C (10 min) hold, then 40 cycles of 95 °C for 15 s and 60 °C for 1 min. Target gene expression was normalized to endogenous controls (actin and GAPDH). C_t_ values were used to calculate ratios of target to control gene using the excel spreadsheet developed by Pfaffl [57].

### 2.5 Transfection

pII cells were seeded at density of 1.5 × 10 [5]. After 24 h of seeding, cells were transfected with 40 nmol of either 200c-miRNA mimic or 50 nmol of miRNA-449a inhibitor using Lipofectamine® RNAiMAX Transfection Reagent (Thermo Fisher Scientific Inc). miRCURY LNA™ miR-200c-3p mimic, and miRCURY LNA™ miR-449a inhibitor was used to transfect the cells, which were also transfected with mimic and inhibitor control (negative control) (EXIQON, USA, Supplementary Table 1). Cells were harvested and pelleted 72 h post transfection. RNA was extracted and assessed, and reverse transcribed into cDNA as already described. Quantitative real time-PCR was performed as described above to measure mRNA quantity to check for either knockdown or over-expression of predicted targets.

### 2.6 Cultrex BME cell invasion assay

Cell invasion was assessed by Cultrex® 24 Well BME cell invasion assay purchased from Trevigen (Cat no. 3455-024-K) according to the manufacturer's instructions. For this, the invasion chamber was coated with 100 μl of 1 x basement membrane extract (BME) solution and incubated overnight at 37 °C. After 24 h transfection, cells were serum starved overnight at 37 °C and 5% CO_2_. On the following day (48 h transfection), cells were harvested, counted, and diluted to 1 × 10^6^ cells per ml in serum-free medium. After that, 100 μl of cells were added to the top chamber of the Cultrex dish. The lower chamber was loaded with 500 μl of DMEM supplemented with 30% FBS (used as a chemoattractant). Cells were incubated at 37 °C, 5% CO_2_ and allowed to invade to the bottom chamber. After 24 h, the top and the bottom chambers were aspirated and washed with 1 x cell wash buffer. Calcein-AM/cell dissociation solution complex was added to the bottom chamber and left for 1 h at 37 °C, 5% CO_2_. Cells internalize Calcein-AM and intracellular esterases cleave the acetomethylester (AM) moiety generating fluorescent free calcein. Invading cells were determined by recording the fluorescence emission using a microplate reader with a filter set of excitation/emission 485/535 nm.

### 2.7 Statistical analysis

In addition to the statistical procedures already mentioned in above sections, Student's two tailed unpaired t-test or one-way ANOVA test followed by Bonferroni post hoc test were used to compare means of individual groups: p < 0.05 was considered statistically significant.

## 3 Results

### 3.1 miRnome sequencing analysis

**Fig. 1:**
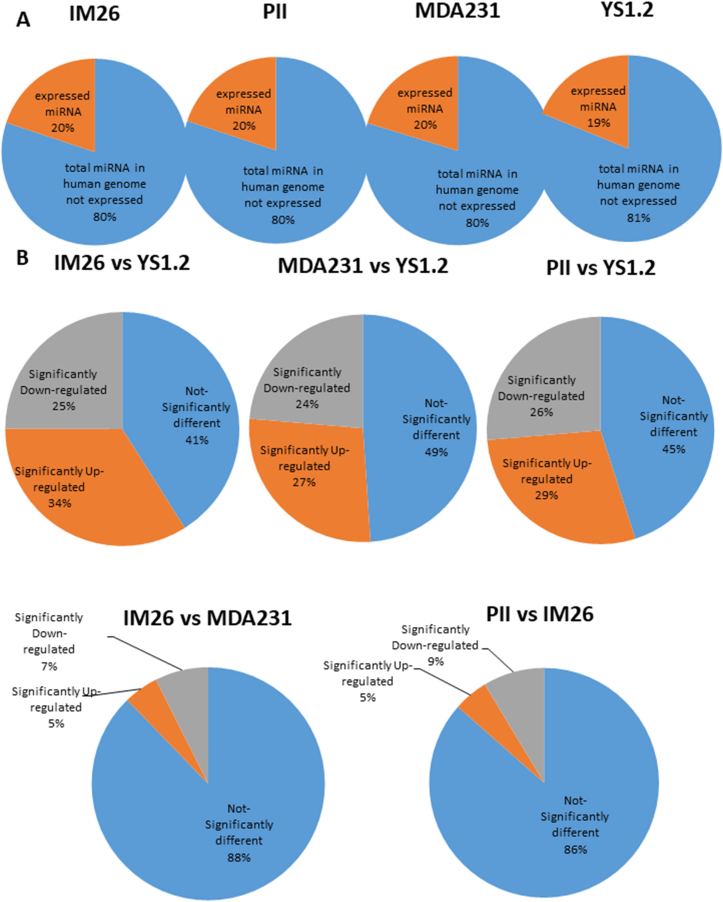
**Percentage of known miRnome expressed in breast cancer cell lines and the miRNAs which are significantly up- or down-regulated when compared between various cell lines**. Panel A: Around 20% of miRNAs are expressed in breast cancer cell lines compared to 2588 total known miRNAs expressed in the human genome. Panel B: the expression profile of miRNAs in ER-was compared with the ER + cell line and between various ER-cell lines. The fold changes were log2-transformed. P-values were calculated as mentioned in methods and significantly differentially expressed miRNA percentages were calculated.

Approximately 20% of the 2588 miRNAs listed in the human miRBase sequences repository available at the time of this study were expressed in individual breast cancer cell lines (Fig. 1 A). Fig. 1 B shows pairwise comparisons between each cell line. For each miRNA the FDR value was calculated to determine significantly differentially expressed miRNAs. Between 27 and 34% of total expressed miRNAs were significantly up-regulated and about 24–26% were significantly down-regulated in the ER-cell lines pII, IM-26 and MDA-MB-231 as compared to the YS1.2. Between the YS1.2 and each ER-cell line there was a difference in the level of expression in 55–59% of expressed miRNAs. In contrast, there was a high degree of similarity in the level of commonly expressed miRNAs between each of the ER-cell lines. Comparison of the significant differences between the *acquired* (pII and IM26) and the *de novo* resistant ER-cell line (MDA-MB-231) showed that they differed in only 12–14% of expressed miRNAs.

**Fig. 2:**
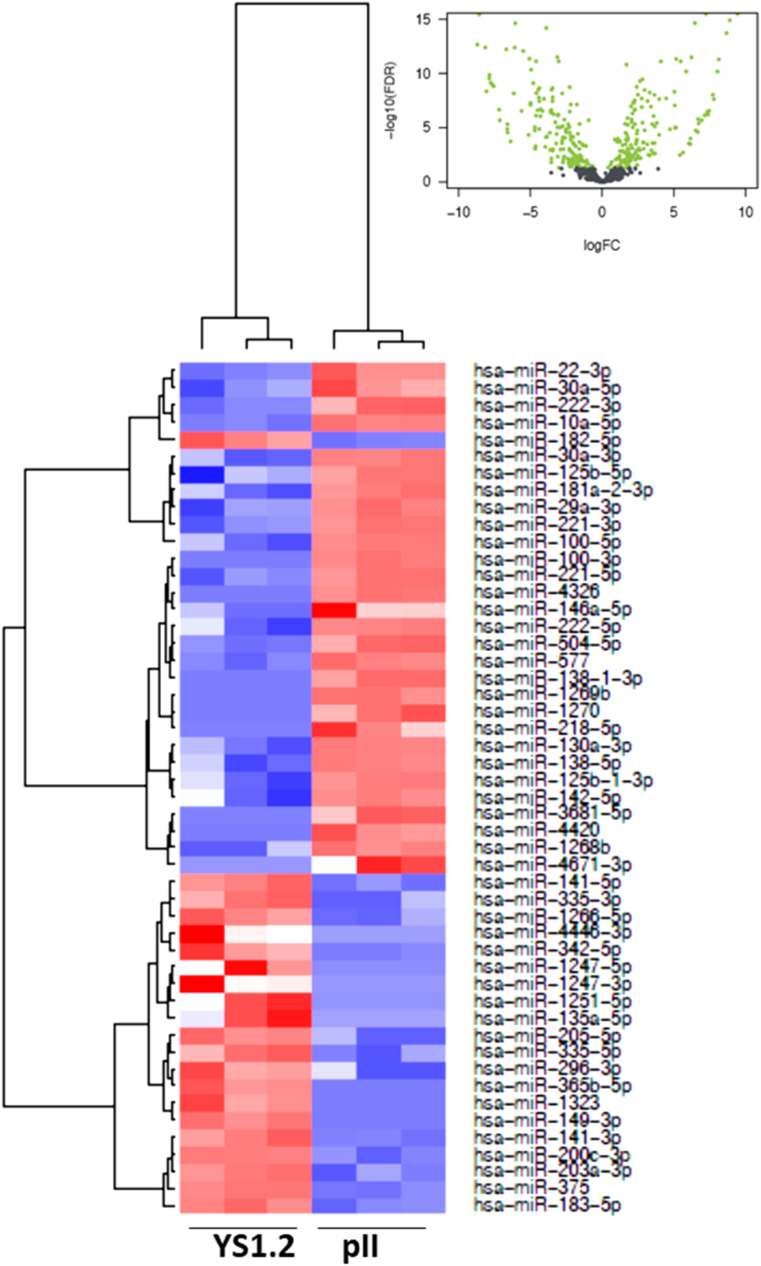
**Pairwise comparison of differentially expressed miRNAs in the ER + YS1.2 and ER-pII breast cancer cell lines**. The volcano plots were generated by plotting the log2 fold change versus –log 10 (P-value) of each comparison. Green points indicate signiﬁcantly differential expressed miRNAs at FDR <0.05. The higher the green point the more significant the difference is and X-axis represents the fold change between the two cell lines being compared. Heat maps were generated in R based on normalized miRNA expression levels of the top 50 most differential expressed miRNAs for each pairing. The blue color represents the down-regulated miRNAs whereas the red color indicates the up-regulated miRNAs. (For interpretation of the references to color in this figure legend, the reader is referred to the Web version of this article.)

**Fig. 3:**
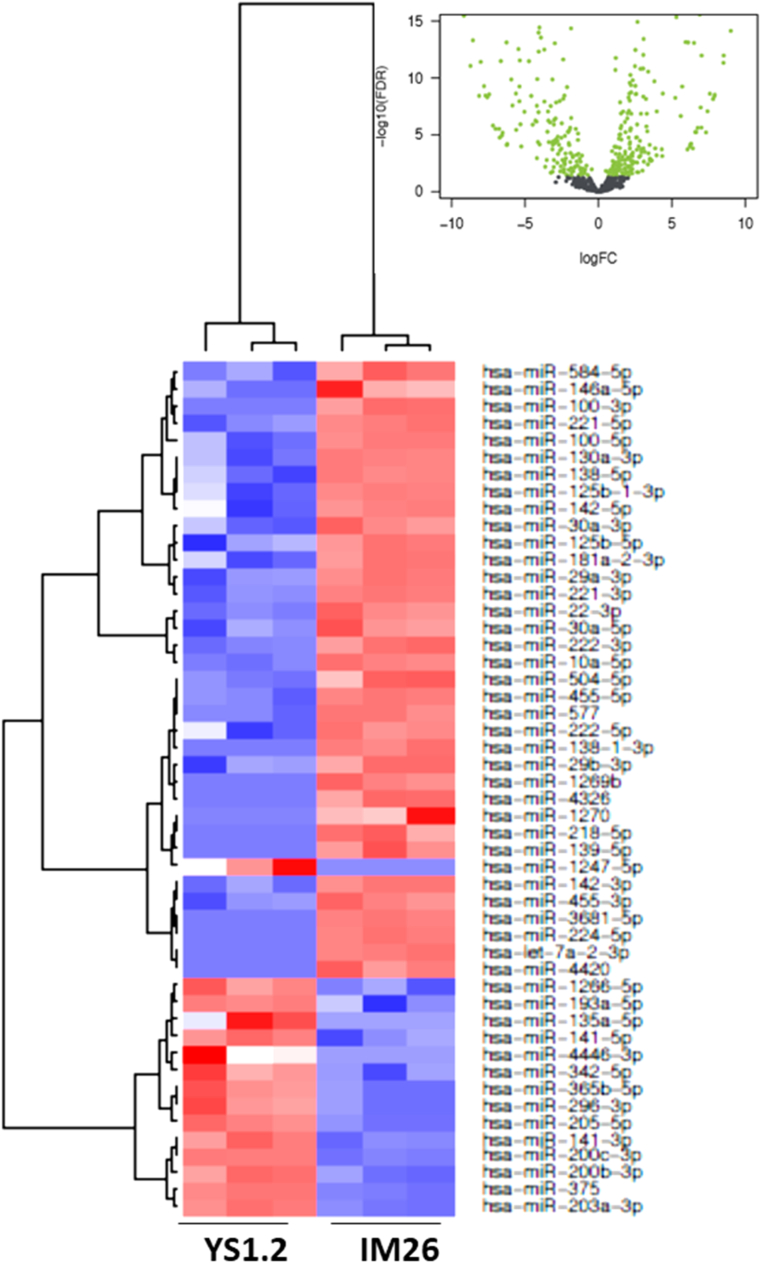
**Pairwise comparison of differentially expressed miRNAs in the ER + YS1.2 and ER- IM-26 breast cancer cell lines**. The volcano plots were generated by plotting the log2 fold change versus –log 10 (P-value) of each comparison. Green points indicate signiﬁcantly differential expressed miRNAs at FDR <0.05. The higher the green point the more significant the difference is and X-axis represents the fold change between the two cell lines being compared. Heat maps were generated in R based on normalized miRNA expression levels of the top 50 most differential expressed miRNAs for each pairing. The blue color represents the down-regulated miRNAs whereas the red color indicates the up-regulated miRNAs. (For interpretation of the references to color in this figure legend, the reader is referred to the Web version of this article.)

**Fig. 4:**
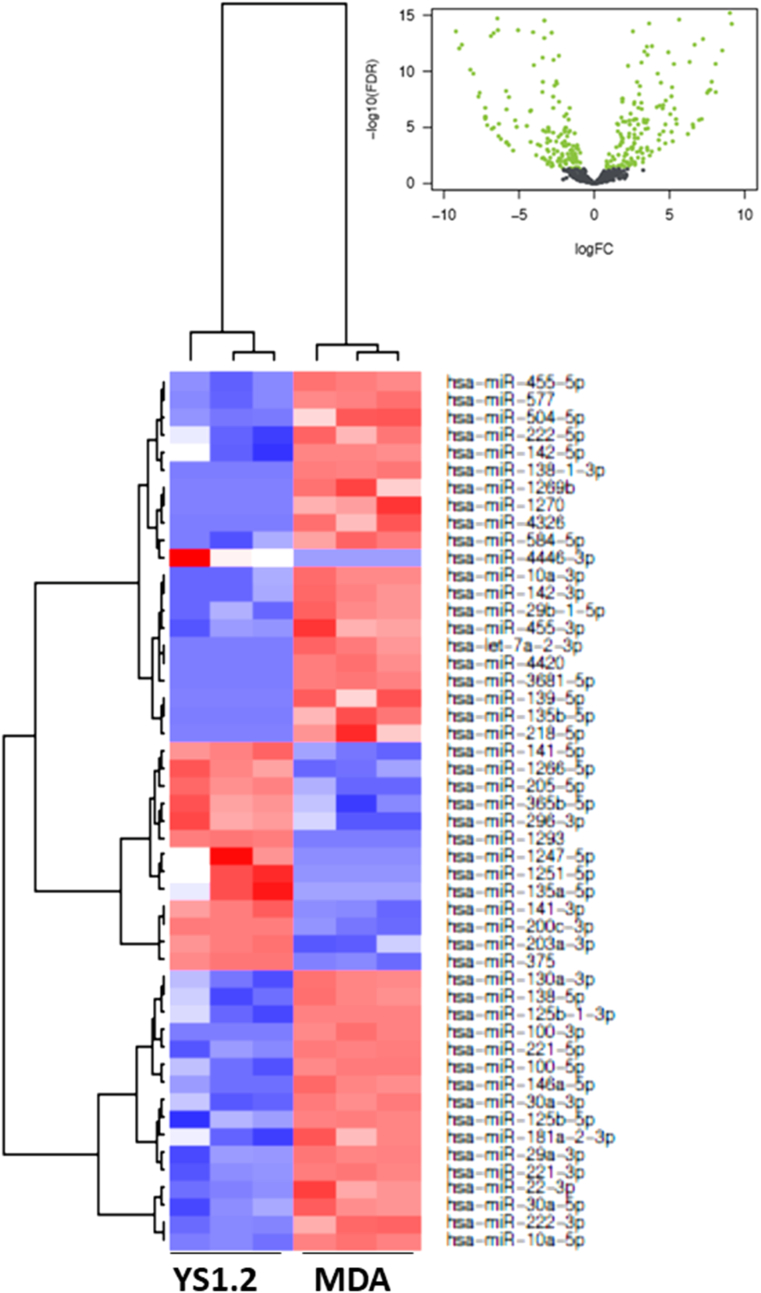
**Pairwise comparison of differentially expressed miRNAs in the ER + YS1.2 and ER- MDA-MB-231 breast cancer cell lines**. The volcano plots were generated by plotting the log2 fold change versus –log 10 (P-value) of each comparison. Green points indicate signiﬁcantly differential expressed miRNAs at FDR <0.05. The higher the green point the more significant the difference is and X-axis represents the fold change between the two cell lines being compared. Heat maps were generated in R based on normalized miRNA expression levels of the top 50 most differential expressed miRNAs for each pairing. The blue color represents the down-regulated miRNAs whereas the red color indicates the up-regulated miRNAs. (For interpretation of the references to color in this figure legend, the reader is referred to the Web version of this article.)

**Fig. 5:**
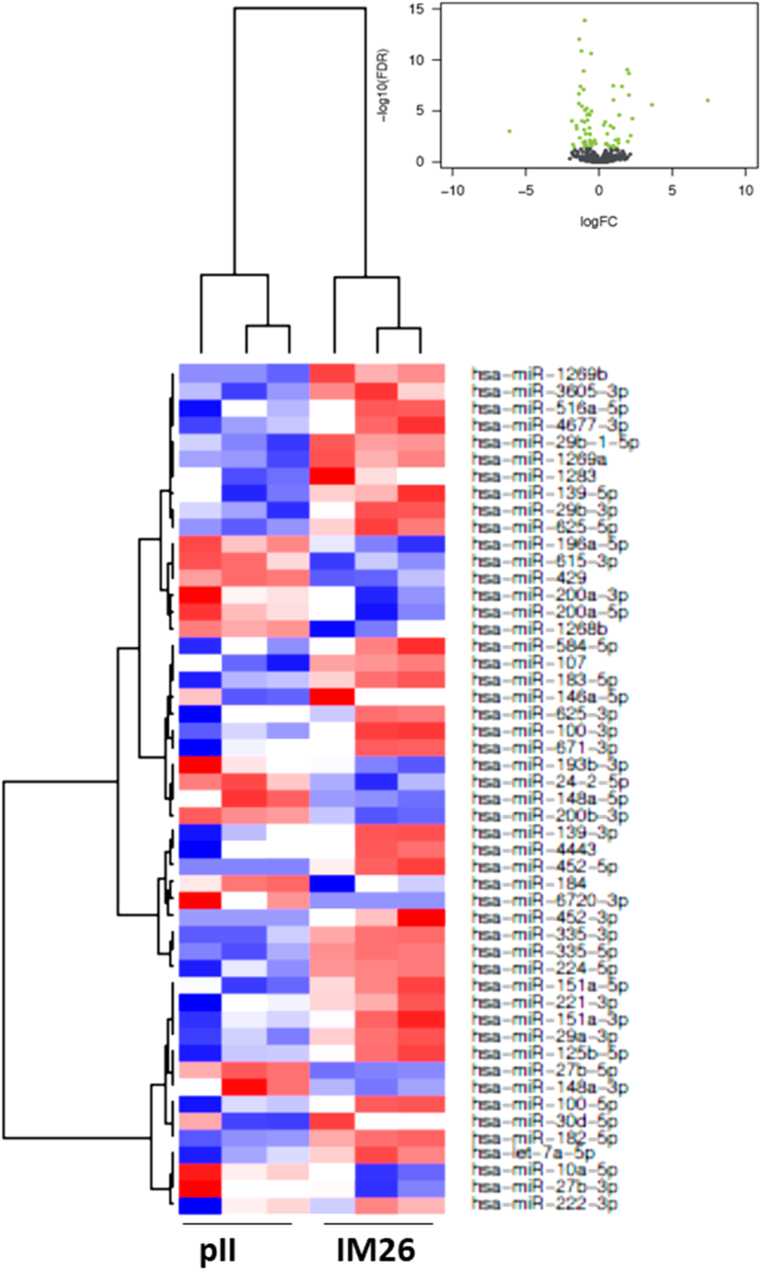
**Pairwise comparison of differentially expressed miRNAs in the ER-pII and IM-26 breast cancer cell lines.** The volcano plots were generated by plotting the log2 fold change versus –log 10 (P-value) of each comparison. Green points indicate signiﬁcantly differential expressed miRNAs at FDR <0.05. The higher the green point the more significant the difference is and X-axis represents the fold change between the two cell lines being compared. Heat maps were generated in R based on normalized miRNA expression levels of the top 50 most differential expressed miRNAs for each pairing. The blue color represents the down-regulated miRNAs whereas the red color indicates the up-regulated miRNAs. (For interpretation of the references to color in this figure legend, the reader is referred to the Web version of this article.)

**Fig. 6:**
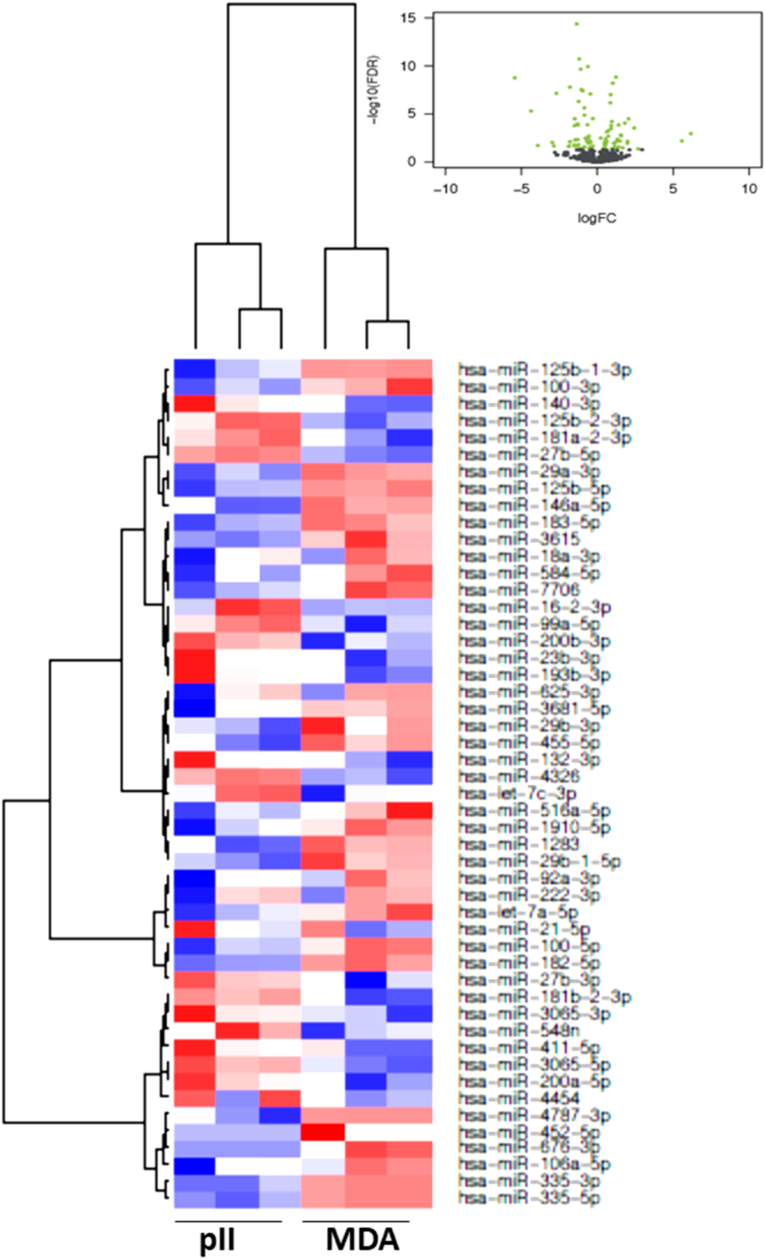
**Pairwise comparison of differentially expressed miRNAs in the ER-pII and MDA-MB-231 breast cancer cell lines.** The volcano plots were generated by plotting the log2 fold change versus –log 10 (P-value) of each comparison. Green points indicate signiﬁcantly differential expressed miRNAs at FDR <0.05. The higher the green point the more significant the difference is and X-axis represents the fold change between the two cell lines being compared. Heat maps were generated in R based on normalized miRNA expression levels of the top 50 most differential expressed miRNAs for each pairing. The blue color represents the down-regulated miRNAs whereas the red color indicates the up-regulated miRNAs. (For interpretation of the references to color in this figure legend, the reader is referred to the Web version of this article.)

**Fig. 7:**
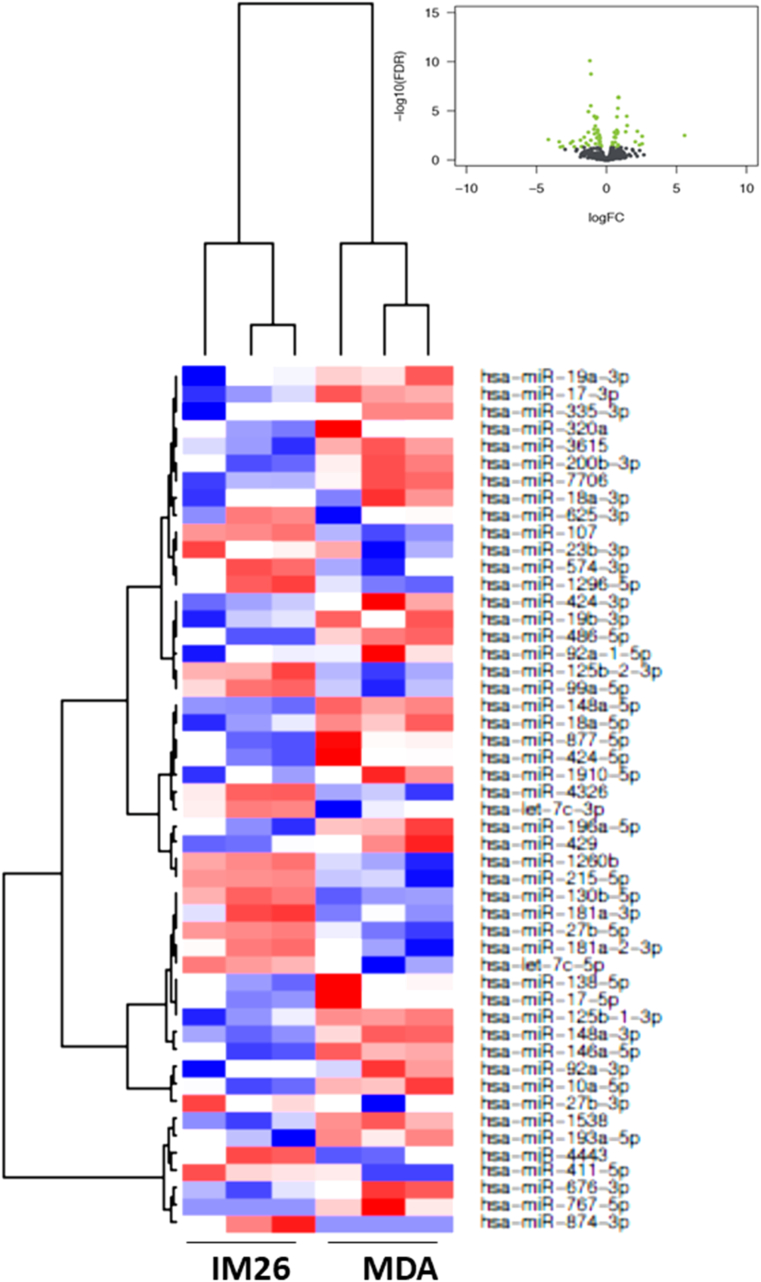
**Pairwise comparison of differentially expressed miRNAs in the ER- IM-26 and MDA-MB-231 breast cancer cell lines.** The volcano plots were generated by plotting the log2 fold change versus –log 10 (P-value) of each comparison. Green points indicate signiﬁcantly differential expressed miRNAs at FDR <0.05. The higher the green point the more significant the difference is and X-axis represents the fold change between the two cell lines being compared. Heat maps were generated in R based on normalized miRNA expression levels of the top 50 most differential expressed miRNAs for each pairing. The blue color represents the down-regulated miRNAs whereas the red color indicates the up-regulated miRNAs. (For interpretation of the references to color in this figure legend, the reader is referred to the Web version of this article.)

In Fig. 2, Fig. 3, Fig. 4, Fig. 5, Fig. 6, Fig. 7, the black colored dots on the volcano plots indicate miRNA's that show no statistical difference between compared populations while the green dots represent those with a significant fold change. In ER-cell lines vs*.* the YS1.2 cell line all volcano plots show similar trends and indicate a high number of significantly differentially expressed miRNAs with wide fold changes. Interestingly, miRNAs do not show the very large differences that are often seen when comparing mRNA levels [45]. Volcano plots that compare the *de novo* resistant cell line with the *acquired* resistance cell lines show differences in expression of much less magnitude. Volcano plots comparing the two acquired resistance ER-cell lines show differences of even less magnitude.

Heat maps with hierarchical clustering were constructed to show the *top* 50 most significantly differentially expressed miRNAs for each comparison. There is substantial similarity in the miRNAs differentially expressed in different comparisons; 38 miRNAs are shared between the three heat maps comparing YS1.2 with IM-26, and pII and MDA-MB-231 respectively. *Acquired* resistance ER-cell lines pII and IM-26 were also compared to the *de novo* resistant MDA-MB-231 cell line. In this case, only 17 differentially expressed miRNAs are shared between the two heat maps. When comparing pII and IM-26 to each other only 4 miRNAs are shared with the two other heat maps that compare *de novo* with acquired resistance lines (miR- 27b-3p, miR-27b-5p, miR-335-3p and miR-625) which indicate that the differentially expressed miRNAs reflect individual differences between the two cell lines.

Table 1, Table 2, Table 3, Table 4 show the detailed characterization of miRNAs based on the degree of fold difference between the different cell lines. It should be noted that many of the significantly differentially expressed miRNAs between the various cell lines do not have a known (i.e., experimentally validated) downstream target related to cancer pathogenesis and we therefore chose miRNAs with known targets related to cancer for subsequent analysis.

**Table 1:** miRs expressed in ER –ve cells only (absent in ER + ve cell).

4–5 fold increase (42)	6–8 fold increase (19)	9–11 fold increase (8)	12–13 fold increase (2)
147b, 3663-3p 486-3p, 488-3p	139-5p, 212-3p	224-5p, 195-5p	100-3p, 221-5p
26a-2-3p, 663b	218-5p, 140-5p	10a-3p, 138-1-3p	
3935, 218-1-3p	142-3p, 132-5p	455-3p, 3681-5p	
29c-5p, 449c-5p	135b-5p, 29a-5p	4326, 4420	
101-5p, 19a-5p	3194-5p, 452-5p		
1304-5p,500a-5p	1270, 1269b		
1262, 2277-3p	598-3p, 766-3p		
664a-3p,1306-3p	590-3p, 29b-1-5p, 1269a, 4774-3p, let7a-2-3p		
4517, 24-1-5p			
4645-3p, 381-3p			
3126-5p, 449a			
4741, 548x-3p			
409-3p, 519a-3p			
3940-3p, 2116-3p			
503-5p, 582-5p			
3152-5p, 651-5p			
4661-3p, 4662-5p			
4671-5p, 4671-3p			
4787-3p, 503-3p			
6852-5p			
let7f-1-3p

**Table 2:** miRs expressed in ER + ve cells only (absent in ER-ve cell).

4–5 fold increase (61)	6–8 fold increase (29)	9–11 fold increase (1)
6738-3p, 4689, 4742-3p, 4700-5p, 4707-5p, 4691-3p	1247-3p, 5680, 4712-3p, 4727-3p, 6833-3p, 4721, 4667-5p, 6785-5p, 203b-3p, 4725-3p, 6850-5p, 196b-5p, 512-3p, 200c-5p	4446-3p
4714-5p, 3691-3p, 6728-3p, 4640-5p, 6729-5p, 4642	1323, 4466, 3190-3p, 4664-3p, 519d-5p, 6860, 6721-5p, 4758-3p, 1247, 5p, 1251-5p	
4526, 4501, 4523, 6777-3p	3074-3p, 4646-5p, 4652-5p, 664b-5p, 6865-5p	
3620-3p, 135a-5p, 3654, 516b-5p 639, 338-5p		
3622b-5p, 431-3p, 6858-5p, 3150b-3p, 1236-5p, 3678-5p		
4426, 6768-5p, 4760-5p, 3159, 6764-5p, 4797-3p		
1252-5p, 1233-3p, 1226-3p, 548b-5p, 548az-5p, 3198		
6747-3p, 6751-3p, 6789-5p, 6795-3p, 6799-3p, 6802-3p		
6807-5p, 6811-5p, 6815-5p, 6818-5p, 6829-5p, 6832-5p		
6834-3p, 6874-5p, 6876-3p, 6877-5p, 6882-5p, 7111-5p		
7151-3p, 7703, 6516-3p

**Table 3:** miRs expressed in ER -ve acquired resistant cells (absent in ER-ve *de novo* resistant).

2–3 fold increase (10)	4–5 fold increase (2)	6–8 fold increase (1)
942-3p, 199a-5p	616-5p, 29b-2-5p	4454
199b-5p, 548j-5p		
1322, 5699-3p		
2682-5p, 3913-5p		
6509-5p, 597-3p

**Table 4:** miRs expressed in ER -ve *de novo* resistant cell (absent in ER-ve acquired resistant).

2–3 fold increase (31)
6812-5p, 6805-5p, 6818-3p, 6514-3p
6500-5p, 6750-3p, 6737-3p, 3617-3p
6764-3p, 4717-3p, 598-5p, 6783-5p
6783-3p, 3529-3p, 5583-5p, 4734
5580-3p, 5587-3p, 4700-5p, 4697-3p
4706, 4440, 676-5p, 3679-5p
3619-5p, 129-2-3p, 3655, 708-5p
1257, 1913, 3188

### 3.2 Experimentally validated target analysis

**Fig. 8:**
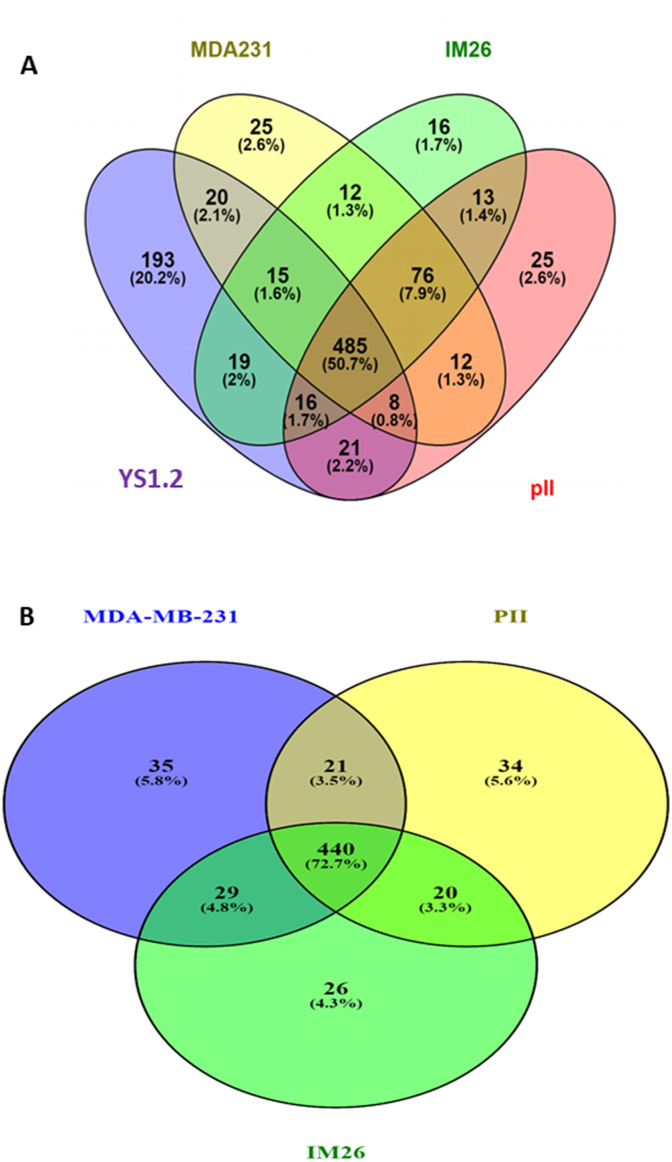
**Venn diagram showing comparison of miRNA between various breast cancer cell lines.** Panel A: The expressed miRNAs in each cell line were plotted using Venny2.1 software as mentioned in methods. 350 miRNAs (51.4% of total) were found to be commonly expressed between all breast cancer cell lines. 13.2% of total miRNAs were expressed only in ER-cell lines and 11.2% only expressed in the ER + cell line. Panel B: The expressed miRNAs in each cell line were plotted using Venny2.1 software as mentioned in methods. 72.7% of total miRNAs are common between the three ER-cell lines with exclusive expression of 4–6% in the individual lines.

Venn diagrams were constructed using the expression of miRNAs in each cell line where the mean of three values was taken for each miRNA. Two diagrams were constructed; one that compares between all the four cell lines (Fig. 8 A) and the second one that compares between the two *acquired* resistance ER-cell lines pII and IM-26, and the *de novo* resistant ER-cell line MDA-MB-231 (Fig. 8 B). A Venn diagram that compares all cell lines shows that they all share about 51% of the total miRNAs that were found to be expressed. All ER-cell lines have less than 3% miRNAs that are exclusively expressed, while the ER + cell line YS1.2 has 20.2% of total miRNAs that are exclusively expressed. Venn diagram that compares ER-cell lines show high similarity (73%) between the three of them. Between pairs there is 76–78% similarity, with only about 4–6% of miRNAs showing exclusive expression.

Expression of Dicer, CTNND2 and ZEB-1.

**Fig. 9:**
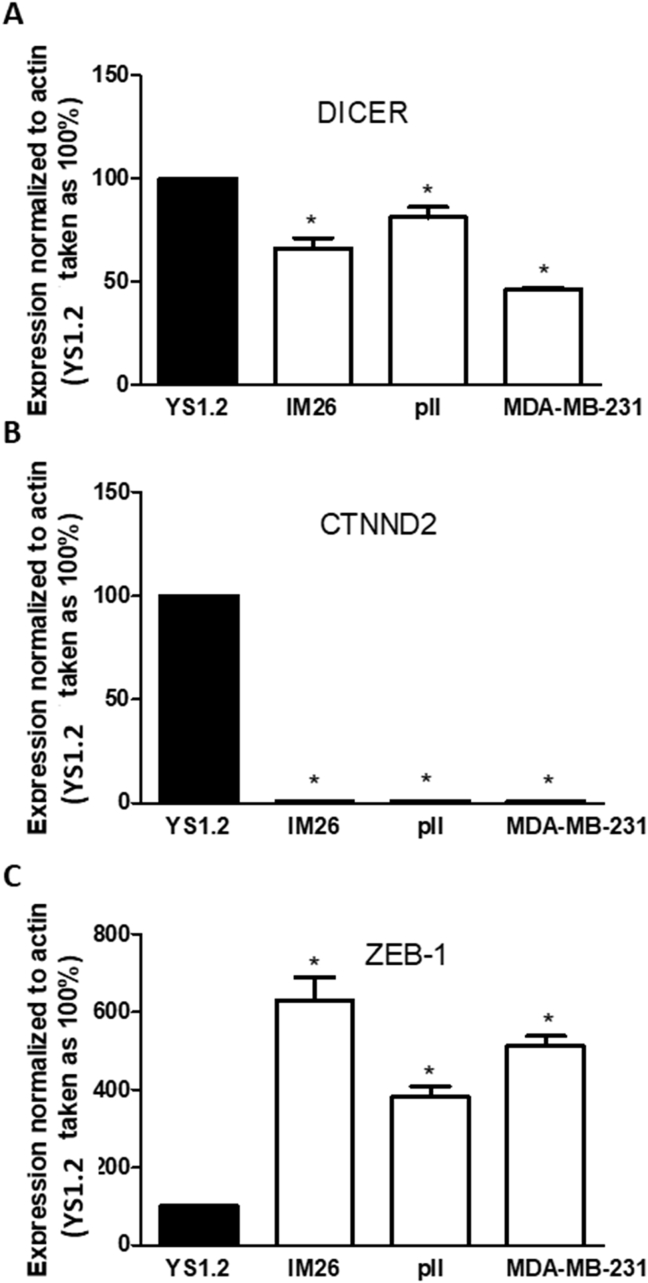
**Expression of dicer, CTNND2, and ZEB1 in breast cancer cell lines**. RNA was extracted from cell monolayers, converted into cDNA and target genes amplified by Taqman real-time PCR as described in Methods. Expression of DICER (A), CTNND2 (B), and ZEB-1 (C) was normalized to YS1.2 cells (set as 100%), with human β actin used as internal control. Histobars are means ± SEM of 3 independent determinations. * denotes significant difference from YS1.2 with p < 0.0001.

There was a significantly lower expression of Dicer in the ER-cells compared to YS1.2 cells (Fig. 9 A). Expression of the epithelial marker CTNND2 was almost abolished in the ER-cells (Fig. 9 B) whereas the expression of the mesenchymal marker ZEB-1 was significantly higher (Fig. 9C).

### 3.3 Transfection of pII cells with miRNA-449a inhibitor and miRNA-200c mimic

**Fig. 10:**
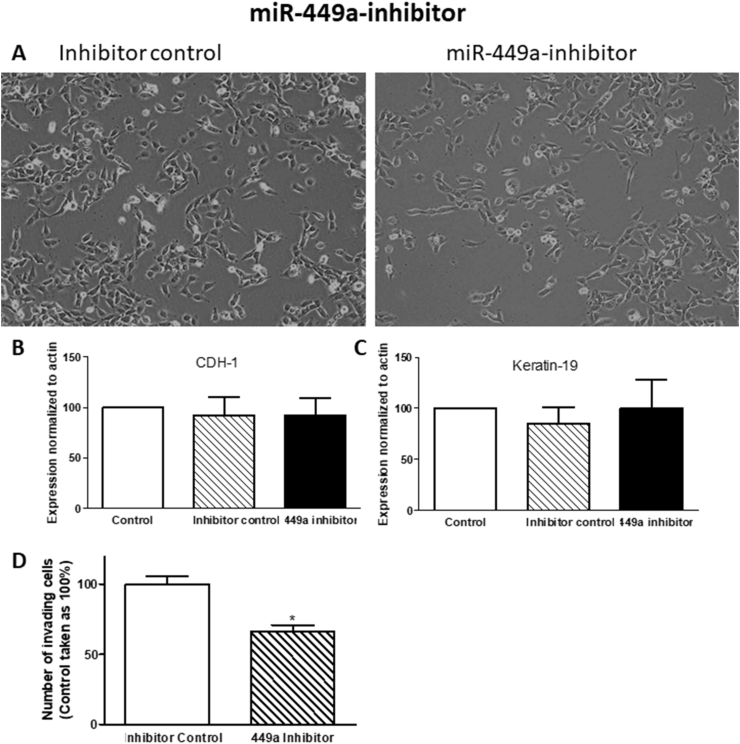
**Effect of miRNA-449a inhibitor treatment on PII cells**. Cells were transfected with inhibitor control or miR-449a inhibitor as described in Methods. Images (20x magnification) were captured using a Leica DFC495 light microscope. Example fields shown were taken 72 h after transfection (panel A). Panels B and C: RNA was extracted from cell monolayers, converted into cDNA and target genes amplified by Taqman real-time PCR as described in Methods. Expression was normalized to control (untreated) cells (set as 100%), with human β actin used as internal control. Histobars are means ± SEM of 3 independent determinations. * denotes significant difference from control with p < 0.0001. Panel D: number of invading cells was determined in pII cells treated with inhibitor control or miRNA-449a inhibitor. Histobars are means ± SEM of 10 independent determinations. * denotes significant difference from inhibitor control with p < 0.05.

**Fig. 11:**
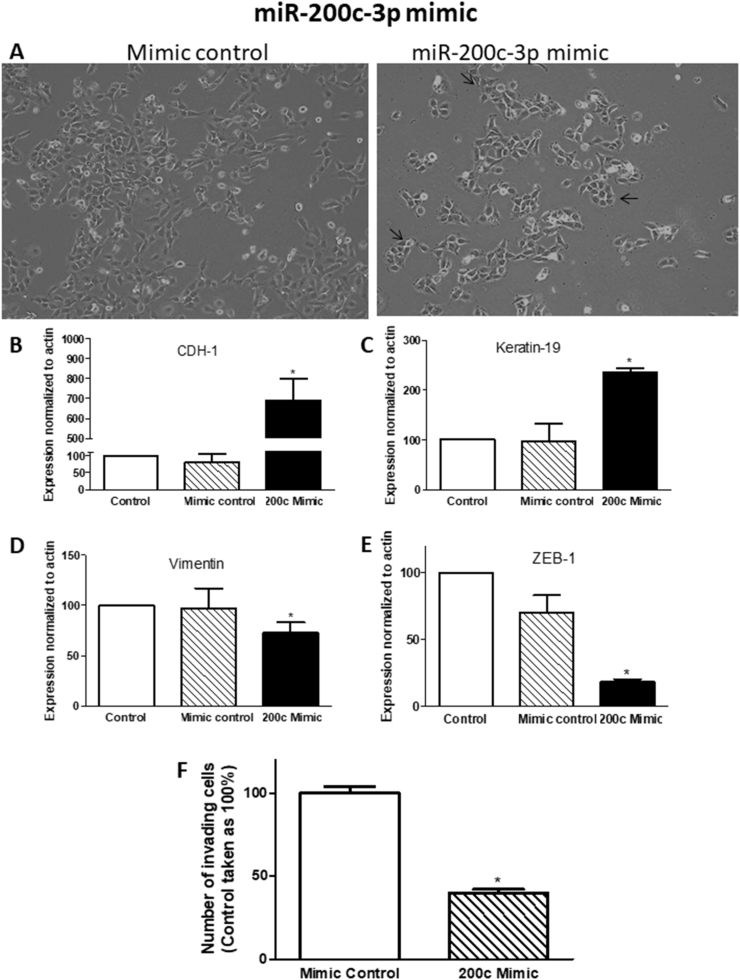
**Effect of miRNA-200c-39 mimic treatment on PII cells**. Cells were transfected with mimic control or miR-200c-3p mimic as described in Methods. Images (20x magnification) were captured using a Leica DFC495 light microscope. Example fields shown were taken 72 h after transfection (panel A). Panels B–E: RNA was extracted from cell monolayers, converted into cDNA and target genes amplified by Taqman real-time PCR as described in Methods. Expression was normalized to control (untreated) cells (set as 100%), with human β actin used as internal control. Histobars are means ± SEM of 3 independent determinations. * denotes significant difference from control with p < 0.0001. Panel F: number of invading cells was determined in pII cells treated with mimic control or miRNA-200c-3p mimic. Histobars are means ± SEM of 10 independent determinations. * denotes significant difference from inhibitor control with p < 0.05.

Cells were transfected with miR-449a inhibitor and miR-200c-3p mimic, and their negative controls, using the protocols described in Methods. Treatment with the miR-449a inhibitor did not change the spindle-like shape of pII cells (Fig. 10 A) and did not modulate the expression profile of the epithelial markers E-cadherin (CDH-1) and Keratin-19 (Fig. 10 B and C). However, it significantly reduced cell invasion by 40% (Fig. 10 D). On the other hand, treatment with the miR-200c-3p mimic resulted in dramatic changes in the shape of pII cells leading to the formation of clusters more resembling YS1.2 morphologies (Fig. 11 A). Also, it significantly up-regulated the epithelial markers CDH-1 and keratin-19 (Fig. 11 B and C), and down-regulated the mesenchymal markers vimentin and ZEB-1 (Fig. 11 D and E) suggesting a reverse mesenchymal to epithelial transition (MET). This also resulted in a significant reduction in cell invasion (Fig. 11 F).

Transfecting pII cell line with the mimic or inhibitor of miR-29a-3p showed no change in epithelial to mesenchymal markers, cell shape, or invasion (data not shown).

### 3.4 Functional analysis and target prediction

For functional analysis targets were either predicted from miRDB database or were searched for as experimentally validated targets in miRTarBase database as mentioned in methods. This is an integrated web server for identifying miRNA-target interactions.

**Fig. 12:**
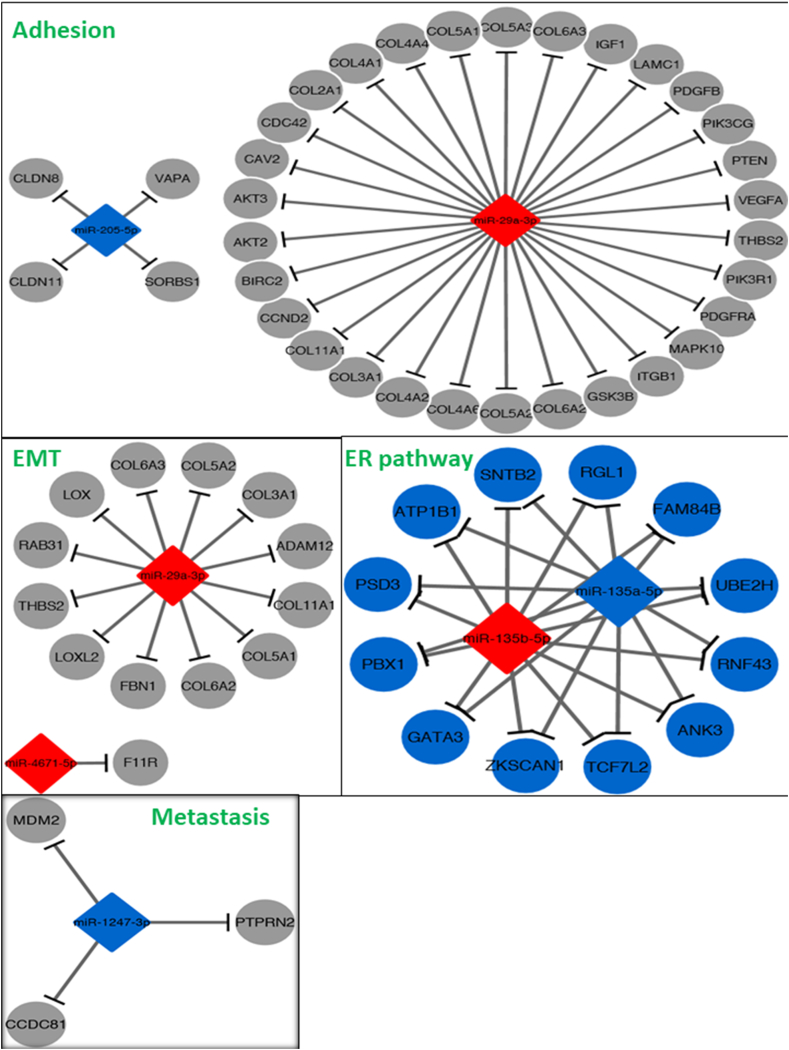
**Network maps showing the miRNAs for which targets are over-represented in gene-sets related to cell adhesion, EMT, estrogen receptor pathway, metastasis, and the respective target genes**. Blue: down-regulated miRNA/gene; red: up-regulated miRNA/gene; gray: gene involved in pathway. Gene-sets were pre-selected from the Molecular Signature Database and multiple Fisher Exact tests were performed as mentioned in methods. (For interpretation of the references to color in this figure legend, the reader is referred to the Web version of this article.)

Gene-sets associated with EMT, metastasis, motility, invasion, estrogen receptor pathway and cell adhesion were selected from the Molecular Signature Database and predicted targets for each miRNA were retrieved from miRDB 5.0. Fisher's Exact test was performed to assess the over-representation of each miRNA's target genes in each of the pre-selected gene-sets explained in methods. Only 7 results were found for all tests which are summarized in the network maps per biological process (Fig. 12). For biological process ‘motility’, no significant results were obtained, while no tests could be performed for the biological process ‘invasion’, as no related gene-sets were available.

## 4 Discussion

Considerable interest has been recently focused on miRNAs as they have been considered potential factors for both breast cancer diagnostics and treatment [58]. In the current study, we aimed to perform miRnome profiling in several breast cancer cell lines with particular emphasis on their ER status. We used the ER-*de novo* resistant MDA-MB-231 and two MCF7 derived lines in which ER had been silenced with shRNA (ER-*acquired* resistant pII, and IM-26), and also a transfected MCF7 line that had failed to down-regulate ER (ER +, YS1.2, positive control for pII and IM-26 cells). Some miRNAs were subsequently selected for further studies on pII cells. We found that approximately 20% of the 2588 known miRNA sequences in the human miRnome was found to be expressed in our breast cancer cell lines. This is a common finding that miRNAs are generally down-regulated in cancer compared to normal tissues [[59], [60], [61]], which is probably due to down-regulation of dicer expression in cancer compared to normal tissues [62]. Also down-regulation of miRNAs is a characteristic of poorly differentiated tumors which offers favorable conditions for cancer cells to proliferate and metastasize [59]. We also found that around 50–60% of the expressed miRNAs were significantly differentially expressed between the three ER-and the ER + cell lines, indicating a substantial difference quantitatively and qualitatively depending on the ER status; most of these significantly differentially expressed miRNAs were up-regulated in ER-cell lines. When we compare between the *acquired* with the *de novo* resistant ER-cell lines (Fig. 2, Fig. 3, Fig. 4, Fig. 5, Fig. 6, Fig. 7), the percentage of significantly differentially expressed miRNAs is only 12–17% indicating great similarity between the two ER-subtypes. The two *acquired* resistant knockout ER-cell lines established in our laboratory have only 14% significantly differentially expressed miRNAs. This is a rather a crucial observation given that these two cell lines were generated quite independently several years apart and yet show such striking similarity, emphasizing the consistency of the consequence of ER knockdown. The relatively few differences may be individual differences arising from random off-target incidents occurring during the separately performed shRNA transfections. Furthermore, the volcano plots that represent significantly differentially expressed miRNAs show that most of the significantly differentially expressed miRNAs exhibit less than 5-fold change when comparing ER-cell lines to each other. On the other hand, the numbers of significantly differentially expressed miRNAs between ER- and ER + cell lines are greatly increased and the numbers of miRNAs that have more than 5-fold change are also increased. It should be noted that the majority of miRNAs only expressed in the ER + cell line inhibits cell proliferation and migration [[63], [64], [65], [66]]. On the other hand, the miRNAs that were down regulated in the ER + cell line and up-regulated in the ER-cell lines contribute to the mesenchymal phenotype. Only a few of the miRNAs that we found over-expressed in the ER-cell lines have been studied functionally in breast cancer and thus their function in ER-cell lines were predicted based on their validated targets. Interestingly, most of the miRNAs that are significantly differentially up-regulated or only expressed in ER-cell lines inhibit cell proliferation, invasion, and migration except for the miR-135 family (miR-135b-3p/-5p), miR-582-5p, miR-221 family and miR-10a-5p. These miRNAs promote cell proliferation, invasion and migration [59,67,68]; miR-582-5p also inhibits apoptosis by targeting apoptotic activation proteins [69].

About 51% of total miRNAs expressed were common in all our breast cancer cell lines indicating the similarity in overall miRNA constitution in breast cancer cell lines which may be used to differentiate between breast cancer and other cancer types when excluding the ones that are common between all cancer types. All ER-cell lines have less than 5% miRNAs that are exclusively expressed in each type, however YS1.2 have 11.2% of miRNAs that are exclusively expressed in this cell line which shows how significantly different the ER + cell line is from ER-cell lines. From the 76 miRNAs that are exclusively expressed in the ER + cell line, only 55 miRNAs are significantly differentially expressed between ER+ and ER-cell lines. On the other hand, from the 90 miRNAs that are commonly expressed in ER-cell lines only 57 miRNAs are significantly differentially expressed. This could be another indicator of how similar the four breast cancer cell lines are. When comparing the three ER-cell lines to each other, all cell lines share about 73% of the total miRNA expressed in each line. Each ER-cell line has less than 6% of their total miRNAs that are exclusively expressed; however, most of them are non-significantly differentially expressed. Only 3 out of 35 miRNAs exclusively expressed in ER-*de novo* resistant cell line is significantly differentially expressed. On the other hand, the only miRNA that is commonly expressed between acquired resistance cell lines and that is significantly differentially expressed is miR-411-5p out of 20 miRNAs. The numbers above serve to emphasize the fact that all the ER-cell lines are of great similarity in their miRNA population and expression.

In regards to miR-200c-3p, it has been previously shown to reverse the EMT process by down-regulating ZEB1 and ZEB2; two of the major regulators of EMT [70]. Other reports showed tumor suppressive properties of the miR-200 family such as inhibiting invasion [71], cell migration [72,73], and metastasis [[74], [75], [76]], and re-sensitization of cells to chemotherapy [76]. In this report, pII cells transfected with miR-200c-3p mimic showed morphological change after 72 h of transfection; many cells showed indication of an EMT reversal with restoration of a more epithelial shape. There was significant increase in expression of epithelial markers E-cadherin and keratin-19. Also, miR-200c-3p mimic down-regulated ZEB1 as has been shown in previous studies [77], this is one of the mechanisms that is thought to be responsible for the reversal of EMT. ZEB1 is a major regulator of EMT as it suppresses the expression of E-cadherin and other cell polarity factors giving rise to the mesenchymal morphology and thereby could promote invasion and metastasis [78]. Suppressing ZEB1 should have the opposite effect; up-regulation of epithelial factors and reversion to an epithelial morphology. Indeed, inhibition of ZEB1 and ZEB2 by miR-200c-3p and miR-200b has been reported to reverse EMT in mesenchymal type MDA-MB-231 and BT-549 cells [77].

About 70 targets are experimentally validated for miR-200c-3p, one of which is BMI1, a protein found to repress PTEN, activate the Akt/GSK3β/Snail pathway and cooperate with Twist to down regulate E-cadherin. Down regulation of BMI1 inhibited EMT [[79], [80], [81]]. Many other genes that contribute to the mesenchymal phenotype such as FN1 (fibronectin 1), NOTCH1 and RHOA [82,83] are also targeted by miR-200c-3p [[84], [85], [86]] explaining the conversion in morphology of mesenchymal to epithelial cell type. Although it might have a positive influence on reversal of EMT, the use of miR-200c-3p as a therapeutic agent remains to be assessed since it has been shown that its over-expression makes a global shift in the proteome, making the cell more metastatic and promotes metastatic colonization through inhibition of the secretion of suppressive proteins [87].

Many studies have linked down regulation of miR-200 and EMT to dicer expression in breast cancer. They have shown that down-regulation of dicer results in down-regulation of miR-200 and subsequently leads to EMT [34,[88], [89], [90]]. This is in agreement with our results which showed reduced dicer expression in several ER silenced cell lines (with mesenchymal cell shape) compared with the ER + cell line YS1.2.

CTNND2 (δ-catenin) is an adhesion protein; a member of the p-120ctn superfamily [91]. Epithelial cells transfected with δ-catenin acquired a more irregular fibroblastic morphology with enhanced cell spreading and cell migration [92]. Although δ-catenin is considered as a potential biomarker for malignancy in breast cancers [93,94], our results showed that it was only expressed in the ER + cell line and not in ER-cell lines. δ-catenin is one of the pro-invasive genes in ER + cell lines and interestingly it increases with tamoxifen treatment while cells are still ER+ [95]. Accordingly, δ-catenin could be a biomarker of only ER + breast cancer invasiveness. Given that δ-catenin is not expressed in ER-cell lines and miR-449a is exclusively expressed in ER-cell lines and that δ-catenin mRNA (CTNND2) is a putative target for miR-449a, we hypothesized that miR-449a targets CTNND2.

In regard to miR-449a, pII cells transfected with miR-449a inhibitor showed no significant change in epithelial or mesenchymal markers. miR-449a was found to target Fos and Met in hepatocellular carcinoma and Flot2 in gastric cancer which was found to reduce features of EMT in these cells [96]. The explanation for our results where there was no conversion in EMT character may be that CTNND2 is independent from EMT in breast cancer or that cells needed a longer time after transfection. Only 18 targets of miR-449a have been experimentally validated in the miRTarBase database [56]. Most of them are oncogenes or promote cell proliferation, such as CDC25A, CDK6, BCL-2, NOTCH1 and E2F3 [[97], [98], [99], [100]]. Two studies have investigated miR-449a in breast cancer; the first showed it suppresses invasion and cell proliferation and that it is regulated by PI3K–C2β; a protein that is related to cancer invasion and metastasis [101]. The second study showed that miR-449a has oncogenic properties in that it targets cysteine-rich protein-2 a transcription factor that inhibits invasion and migration [102]. We have confirmed that miR-449a targeted CTNND2, whose overexpression increases invasion and malignancy.

Thus, in summary, in this study we have shown that miR-449a could be considered as a tumor suppressive miRNA in ER + tumors, and that miR-200c, which targets ZEB1 and ZEB2 (major regulators of EMT) can reverse EMT in our cell line pII an ER-acquired resistance cell line. miR-29a-3p needs to be further investigated. Taken together, the net action of miRNAs determines the cell phenotype. Apparently, most of the miRNAs work as tumour suppressers rather than oncogenes since in both ER- and ER + cell lines they inhibit cell proliferation, invasion, and metastasis. This would be consistent with their lower expression in cancer cells. It seems that the reduction of the total miRNA in breast cancer is a mechanism to increase cancer ability to metastasize and invade other tissues. It would be useful to compare miRnome profiling with proteomics to determine the optimal combination of miRNAs that could be introduced or inhibited for targeted therapy. From our analysis we can conclude that EMT can be regulated by miRNAs by targeting mRNAs that are important in EMT regulation, and to re-sensitize endocrine resistant breast cancers by turning them back into a type that will be susceptible to endocrine agents [103]. The miRNAs selected for the transfection experiments in this study were used just an example and to confirm previous findings, using our endocrine resistant breast cancer cell lines (pII and IM-26), which were generated through ER knockdown. Also, these findings suggest that the other miRNAs that we have identified as differentially regulated would be worth investigating in future studies.

## Author contribution

Conceived and designed the experiments: MAK, YAL. Performed the experiments: AA, MAK. Analyzed the data: YL, MAK, AA. Contributed reagents/materials/analysis tools: YL, MAK. Wrote the paper: MAK, YAL, AA.

## Declaration of competing interest

The authors confirm that there is no conflict of interest.

## Data Availability

Data will be made available on request.
