# Supplementary material for: MicroRNA expression profiling of endocrine sensitive and resistant breast cancer cell lines
Source: Biochem Biophys Rep. 2022 Jul 20;31:101316. doi: 10.1016/j.bbrep.2022.101316 (PMC9307586; doi:10.1016/j.bbrep.2022.101316)
Supplement: Multimedia component 1 [file mmc1.docx]

Supplementary Table 1. Sequences of ER shRNA and miRNA mimics

| ER shRNA | 5'GGATCCCGTATGGCTATGGAATCTGCTTCAAGAGAGCAGATTCCATAGCCATACTTTTTTCCAAAAGCTT-3′  \|   Sense   \| Loop   \| antisense   \| Termination Signal    cloning sites are BamH1 and Hind111 in vector pRNA-U6.1/Neo (SD1201) from GenScript |
| --- | --- |
| hsa-miR-200c-3p miRCURY LNA miRNA Mimic Exiqon | MIMAT0000617: 5'UAAUACUGCCGGGUAAUGAUGGA-3′ |
| hsa-miR-29a-3p miRCURY LNA miRNA Mimic Exiqon | MIMAT0000086: 5'UAGCACCAUCUGAAAUCGGUUA-3′ |
| hsa-miR-449a miRCURY LNA miRNA MimicExiqon | 5′-ACCAGCUAACAAUACACUGCCA-3′ |
| Negative Control miRCURY LNA miRNA Mimic Exiqon | microRNA strand: UCACCGGGUGUAAAUCAGCUUG |

Note: miRNA inhibitor sequences were not provided by Exiqon
